# Distribution characteristics of pathogens in different stages of pressure ulcers and the therapeutic effect of linear polarized polychromatic light combined with silver sulfadiazine cream

**DOI:** 10.1097/MD.0000000000035772

**Published:** 2023-10-20

**Authors:** Binxiong Chen, Yang Liu, Yueming Liu, Shi Xu

**Affiliations:** a Department of Burn and Plastic Surgery, Shenzhen Longhua District Central Hospital, Affiliated Central Hospital of Shenzhen Longhua District, Guangdong Medical University, Shenzhen, Guangdong, China; b Department of Medical Laboratory, Shenzhen Longhua District Central Hospital, Affiliated Central Hospital of Shenzhen Longhua District, Guangdong Medical University, Shenzhen, Guangdong, China.

**Keywords:** linear polarized polychromatic light, pressure ulcers, silver sulfadiazine cream, wound healing

## Abstract

To investigate the distribution characteristics of pathogens in different stages of pressure ulcers and observe the application of linear polarized polychromatic light (LPPL) combined with silver sulfadiazine cream in treating varying stages of pressure ulcers. This study comprised 88 patients with pressure ulcers who were enrolled in the department of burn and plastic surgery of our hospital from April 2019 to April 2022. The wound exudates from patients were collected, followed by analyzing the distribution of pathogens in different stages of pressure ulcers. Patients were randomly divided into 2 groups. The first group (n = 44) received LPPL combined with silver sulfadiazine. The other group was intervened with LPPL group only for 2 weeks. The clinical efficacy, condition, and pain in the 2 groups, as well as the healing timeframes for patients were measured at different stages. The findings showed that among 88 patients with pressure ulcers, 62 were infected, and the infection rate was 70.45%. The pathogens that were observed in stage II and III to IV pressure ulcers were mainly Gram-negative bacteria. The total effective rate in the combined group was 90.91%, which was much higher than that of LPPL group (70.45%). Compared with LPPL group, the pressure ulcer scale for healing and visual analogue scale scores in the combined group were markedly lower (*P* < .05). It is important to note that in LPPL group, the healing time of patients in stage II and stage III to IV in the combined arm were 9.76 ± 2.38 days and 13.19 ± 2.54 days, respectively. The corresponding time in the LPPL group was prolonged to 13.20 ± 3.76 and 18.82 ± 4.17 days, respectively. The main pathogens associated with wound infection in patients with pressure ulcers are Gram-negative bacteria. The curative effects of LPPL combined with sulfadiazine silver cream on patients with pressure ulcer is obviously improved, and the recovery and pain relief are faster while the healing time of pressure ulcer is shorter.

## 1. Introduction

Pressure ulcers chronic refractory wounds that are characterized by high incidence, rapid development, and easy infection. They are difficult to cure, thereby posing a heavy social burden for health care system.^[1]^ The pooled prevalence estimates for pressure ulcers within hospitalized adults was 12.8% and the incidence rate was 0.054% days worldwide. Pressure ulcers affect approximately 3 million adults in the United States.^[2,3]^ Several extrinsic and intrinsic factors are attributed to the development of pressure ulcers, and these include ischemia, decreased autonomic control, older age, anemia, malnutrition, muscle stretches, friction, and moisture.^[4]^ The National Pressure Ulcer Advisory Panel made the latest classification descriptions on pressure ulcers, which gave rise to 6 types as follows: stage I (non-blanchable erythema), stage II (partial-thickness damage), stage III (full-thickness skin loss), stage IV (full-thickness tissue loss), uninstallable, and suspected deep tissue injury stage. The different stages of pressure ulcer progression were determined based on different pathological characteristics.^[5]^ Currently, the clinical treatment of pressure ulcers remains challenging, further emphasizing the need for multiple interventions. For pressure ulcers in stage I or stage II, conventional wound treatments were available to improve the basic physiological state of the situation. The commonly used conservative treatment options include pressure relief, local wound care, negative pressure wound therapy, debridement, and nutrition.^[4]^ However, these conventional treatments have limited effects on pressure ulcers in stage III or stage IV, which might require surgical management. Though surgical reconstruction may allow patients to recover, resume their daily activities, and enjoy a better quality of life, it has potential risks. All patients who receive surgical reconstruction might have an extended recovery, both physically and psychologically. Moreover, the patients might have to face indefinite outpatient surveillance, in addition to being exposed to the risk of recurrence.

As a topical antibiotic medication, the silver sulfadiazine cream has been widely applied in preventing and treating wound infections in patients with partial-thickness burns or venous stasis ulcers.^[6]^ Currently, a myriad of novel modified dressings of silver sulfadiazine have been developed to improve the antibacterial and healing effects.^[7,8]^ Silver sulfadiazine-loaded core-shell nanofibers are effective in enhancing burn wound healing.^[9]^ Honey-based silver sulfadiazine micro-sponge that is loaded with hydrogel can serve as a potential alternative for treating burn wounds.^[8]^ However, the conventional dressing of silver sulfadiazine might adhere to the wound surface. Compounds that contain silver tend to delay the wound healing process, considering that silver may have severe cytotoxic activity in various host cells.^[10]^ Therefore, new clinical managements are required to overcome the shortcoming of silver sulfadiazine.

The therapeutic effects of phototherapies have been documented for several decades with clinical applications that include neonatal jaundice, psoriasis, and vitiligo.^[11,12]^ Linearly polarized light belongs to a physical therapy method, which combines electronic technology and infrared technology to improve local inflammation through photoelectric reaction and photothermal reaction. As one of the classical phototherapies, linear polarized polychromatic light (LPPL) was widely clinically applied for dealing with conditions ranging from skin ulcers, burns, and chronic wounds, to musculoskeletal injuries.^[13–15]^ The biomedical effects of LPPL were well-recognized including improving local microcirculation, promoting collagen regeneration, angiogenesis and keratinosis, enhancing immunomodulatory effects, and antibacterial action.^[16]^ However, the possibility that LPPL could improve the antimicrobial efficiency of silver sulfadiazine in clinical practice is still unclear. In this study, the therapeutic effects of LPPL combined with silver sulfadiazine cream were explored on different stages of pressure ulcers, in a bid to provide a basis for the treatment of pressure ulcers.

## 2. Data and Methods

### 2.1. General information

The participants in this study were selected from pressure ulcer patients who were admitted to the department of burn and plastic surgery in our hospital, from April 2021 to April 2022. This study was approved by the Institutional Review Board of Shenzhen Longhua District Central Hospital, Guangdong Medical University. The inclusion criteria were: Patients with Phase II to IV ulcer according to the National Pressure Ulcer Advisory Panel classification system; Pressure ulcers on the sacrum, ischial tubercle, hip, and heel; Absence of relative contraindications for using LPPL and silver sulfadiazine cream; The patient was informed of the study. The exclusion criteria were: The presence of serious skin tissue lesions; Unstable blood glucose levels; If hormone drugs and immunosuppressants were used within 1 month; Those physically poor and cannot turn over independently. A total of 88 patients were randomly divided into a combined cream group (n = 44) and LPPL group (n = 44). The general data of the 2 groups are displayed in Table 1.

**Table 1:** Comparison of two groups of general information.

		Combined cream group (n = 44)	LPPL group (n = 44)	*t/χ^2^/Z*value	*P* value
Gender [n (%)]	Male	23 (52.27)	24 (54.55)	0.046	.831
	Female	21 (47.73)	20 (45.45)		
Age (yr)		69.11 ± 6.54	70.03 ± 6.17	0.679	.499
Pressure sore area (cm^2^)		18.28 ± 4.12	18.01 ± 4.55	0.292	.771
Stage of pressure ulcer [n (%)]	Stage II	27 (61.36)	28 (63.63)	0.352	.839
	Stage III	15 (34.09)	15 (34.09)		
	Stage IV	2 (4.55)	1 (2.27)		
Pressure sore site [n (%)]	Sacrum	18 (40.91)	20 (45.45)	1.495	.683
	Ischial tubercle	10 (22.73)	7 (15.91)		
	Hip	11 (25.00)	14 (31.82)		
	Heel	5 (11.36)	3 (6.82)		

LPPL = linear polarized polychromatic light.

### 2.2. Methods

#### 2.2.1. Sample collection and pathogen determination.

A sterile cotton swab soaked with physiological saline (0.9%) was used to wipe the pressure sore surface, and secretions in the depths of the wound or fistula were collected using another swab. A sterile test tube was employed for storing the sample for inspection, after which the automatic microbial identification system was used to identify the pathogens.

#### 2.2.2. Interventions.

The patients in both groups received active treatment for underlying diseases. Nutritional support and regular body-turning training were provided according to surgeon’s advice. The ward was well-ventilated and the bed sheets were kept dry. Pressure ulcers in different stages were treated with corresponding cleaning, disinfection, and debridement.

LPPL was applied using Super Lizer (HA-2200 LE1, Tokyo Iken Co. Ltd). A linear polarized light source (Bioptron lamp) with the following technical parameters was used: energies delivered were typically 4 J/cm^2^ per minute and degree of polarization > 95%. LPPL was performed twice daily, at a distance of 1 cm, 5 times a week.

The combination group was treated with the sulfadiazine silver cream (Kunming China Resources Shenghuo Pharmaceutical Co., Ltd., GYZZ H20057720) on the basis of the LPPL group. The sulfadiazine silver cream was applied on the pressure sore surface with a thickness of about 2 mm, before being wrapped and fixed with a sterile gauze. According to the exudate, the dressing was changed once every 2 to 3 days, for 2 weeks.

### 2.3. Observation indicators

#### 2.3.1. Distribution of pathogenic bacteria.

The pathogens in patients were counted, and the distribution characteristics and constituent ratio of the infection were analyzed.

#### 2.3.2. Evaluation standard.

The evaluation standards were as follows: Cure (local tissue repair, scab on the sore surface); Effective (the scope of the sore surface is reduced by more than 50%, some scabs, and no exudates and secretions); Ineffective (there are still exudates and secretions on the sore surface); Worsening (the area of the sore is enlarged). The total effective rate was calculated using the formula.


$$\begin{array}{l} Total\;ef\;fective\;rate=Cure\;rate+Ef\;fective\;rate. \end{array}$$

#### 2.3.3. Evaluation of healing and pain.

The patient’s condition was evaluated using the pressure ulcer scale for healing (PUSH) tool before and after treatment. The tool includes 3 dimensions, which are tissue type, scope, and exudate amount, corresponding to 0 to 4 points, 0 to 10 points, 0 to 3 points, respectively. Each parameter is scored and the total possible score ranges from 0 to 17 points. The high scores indicate serious conditions of the wounds.^[17]^ The visual analogue scale (VAS) tool was widely used to evaluate pain intensity. This tool is a subjective evaluation scale, by which the patients’ feeling of pain is marked on a straight line of 0 to 10 cm. Generally, 0 points represent no pain while 10 points represent unbearable pain.

#### 2.3.4. Healing time of patients at different stages.

The tissue morphology, range, and exudate of the 2 groups were dynamically observed. The wound healing time of patients was recorded. The healing time in stages II to IV was statistically analyzed.

### 2.4. Statistical methods

The SPSS 23.0 (SPSS, Chicago, IL) was applied for analyzing the data, and the age, pressure sore area, PUSH, VAS score, and other measurement data were displayed as mean ± standard deviation (*x̄* ± sd). The independent sample *t* test or paired sample *t* test was performed between groups or within groups. The number of cases and the rate (%) were applied to calculate the site of pressure ulcers, as well as the effective rate. The *χ2* test was performed to enhance comparisons between groups. The stage of pressure ulcer was grade data, and a rank sum test was performed between groups. The *P* value < .05 was considered statistically significant.

## 3. Results

### 3.1. Distribution characteristics of pressure sore pathogens in different stages

Among the 88 patients with pressure ulcers, 62 cases were complicated with pathogen infection. These included 27 cases of stage II pressure sore and 35 cases of III to IV stage pressure ulcers with infection, with an infection rate of 70.45% (62/88). In patients with stage II pressure sores that were complicated with infection, a total of 27 strains of pathogenic bacteria were isolated, including 19 strains of Gram-negative bacteria (70.37%) and 8 strains of Gram-positive bacteria (29.63%). A total of 35 strains of pathogenic bacteria were isolated from patients with co-infection in the III to IV stage, including 23 strains of gram-negative bacteria (65.71%), 11 strains of Gram-positive bacteria (31.43%) and a strain of fungi (2.86%).

### 3.2. Clinical efficacy

The number of cured cases in the combined cream group was more than that in the LPPL group. Moreover, the total effective rate in the combined cream group was 90.91%, which was markedly higher than that in the LPPL group (*P* < .05), as shown in Table 2.

**Table 2:** Clinical efficacy [n (%)].

Group	Cure	Effective	Ineffective	Worsening	Total effective rate
Combined cream group (n = 44)	32 (72.73)	8 (18.18)	4 (9.09)	0 (0.00)	40 (90.91)
LPPL group (n = 44)	20 (45.45)	11 (25.00)	11 (25.00)	2 (4.55)	31 (70.45)
*χ^2^* value					5.906
*P* value					.015

LPPL = linear polarized polychromatic light.

### 3.3. Evaluation of condition and pain

As shown in Table 3, the PUSH and VAS scores of the combined cream group were markedly lower than those of the LPPL group (*P* < .05), after treatment.

**Table 3:** Evaluation of condition and pain (`x ± s, points).

Group	PUSH		VAS	
	Pretreatment	Post-treatment	Pre-treatment	Post-treatment
Combined cream group (n = 44)	9.17 ± 2.23	4.42 ± 1.15*	6.17 ± 1.12	1.53 ± 0.38*
LPPL group (n = 44)	9.11 ± 2.15	5.24 ± 1.24*	6.12 ± 1.04	1.89 ± 0.41*
*t* value	0.128	3.216	0.217	4.272
*P* value	.898	.002	.829	<.001

Compared with before treatment.

LPPL = linear polarized polychromatic light, PUSH = pressure ulcer scale for healing, VAS = visual analogue scale.

* *P* < .05.

### 3.4. Healing time of patients at different stages

The healing time of patients in different stages such as stage II, III to IV in the combined cream group was shorter than that of the LPPL group (*P* < .05), as shown in Table 4.

**Table 4:** Healing time of patients at different stages (`x ± sd).

Group	Stage II	Stage III–IV
Combined cream group (n = 44)	9.76 ± 2.38	13.19 ± 2.54
LPPL group (n = 44)	13.20 ± 3.76	18.82 ± 4.17
*t* value	5.128	7.649
*P* value	<.001	<.001

LPPL = linear polarized polychromatic light.

## 4. Discussion

Currently, pressure ulcer management is still one of the most challenging clinical issues. Variations in patients conditions and ulcer management lead to tough systematic clinical observations. The aim of the present study was to evaluate the effects of the combination of LPPL and SSD in the healing process of pressure ulcers. Our study displayed that the clinical efficacy of the combination arm was higher than that of LPPL group. Besides, the PUSH, VAS scores, and healing time were also supportive of the notion that the combination of LPPL and SSD can improve the treatment efficacy for pressure ulcers. Pressure ulcers occur due to a shortage of blood supply and removal of metabolites when the external pressure exceeds capillary blood pressure over a long time.^[18]^ The occurrence of pressure ulcers is associated with many factors, including the external ones like humidity and soft tissue mechanical force, as well as internal factors such as basic diseases, fever, infection, anemia, malnutrition, and endothelial cell dysfunction.^[19]^ The elderly frail people are the high-risk group for pressure ulcers because they often complications of basic diseases and are more vulnerable to infections, even when effective care is given.

In this study, patients with pressure ulcers from stage II to IV were included, and it was found that the wound infection rate was 70.45%. Among the pathogens of wound infection in the different stages of pressure sores (stage II and III–IV), Gram-negative bacteria accounted for the highest proportion, followed by Gram-positive bacteria, suggesting that gram-negative bacteria were the dominant pathogens in pressure sore wounds, and this finding was consistent with previous investigations.^[20]^ It is important to note that a complex microbiome is a hallmark of pressure wounds and commensal bacteria might trigger a positive cutaneous immune response to prevent wound infections.^[21]^ However, because of the nature of the study design, the evolution of bacterial microbiota was missed, which could provide clear evidence for changes in diversity and the composition of the microbiota.

In this study, LPPL combined with silver sulfadiazine cream was used. It was confirmed that the total effective rate of the combined cream group was markedly higher; the PUSH and VAS scores were markedly lower; and the healing time of patients with stage II, III, and IV was markedly shorter. The aforementioned results suggest that the therapeutic effects of LPPL combined with the silver sulfadiazine cream are be superior to those of LPPL alone, which can relieve pain and promote the healing of pressure ulcers at different stages. Previous studies suggest that the advantage of LPPL is that it can penetrate the skin, with a depth of up to 5 cm, and reach the deep tissues, thus enhancing wound healing and immunomodulatory effects.^[22]^ Linearly polarized light has been shown to accelerate the healing process of ulcers, surgical wounds, skin burns, and minor musculoskeletal injuries.

Previous studies also mentioned that LPPL could trigger the human cellular and humoral defense, and rearrange the polar heads of the lipid bilayer in cell membranes thus enhance enzyme reactions.^[14]^ Despite of mounts of studies that demonstrate the positive effects of LPPL, the underpinning mechanism of its biological effects is not adequately articulated.^[23]^ Of note, light intensity leads to mixed effects on cellular viability, with a fluence of 0.5 to 5.5 J/cm^2^ to promote cell viability, the fluence of 1.5 to 25 J/cm^2^ demonstrated little effect, while that of 1.5 to 25 J/cm^2^ displayed inhibition on cell viability.^[24]^ The sulfadiazine silver cream exhibits the pharmacological effects of both sulfadiazine and silver salt. Sulfadiazine can inhibit the growth and reproduction of Gram-positive and Gram-negative bacteria by inhibiting the dihydrofolate synthase. The silver salt can dry the wound, astringe it, and promote wound healing.^[25,26]^ In order to compare the healing time of full-thickness wounds that are treated with local estrogen, phenytoin, or sulfadiazine silver, Mirnezami and colleagues conducted an in vivo study on 32 male Wistar rats. The experiments demonstrated that the average healing times in estrogen, phenytoin, SSD, and control groups were 11, 10, 7.62, and 11.87 days, respectively. The wound healing speed of the sulfadiazine silver group was markedly faster than that of phenytoin and estrogen groups.^[27]^ A number of reports illustrated the efficacy of hyaluronic acid and 1% sulfadiazine silver in the process of wound healing retrospectively. The results showed that after sulfadiazine silver treatment, wound size and the inflammatory process were reduced. Moreover, the proliferation of bacterial colonies on the wound was reduced in a dose-dependent manner. The researchers believed that the combined remedy of 1% sulfadiazine silver and hyaluronic acid could promote wound healing.^[28,29]^ However, the side effects of sulfadiazine silver could not be overlooked. Although this heavy metal topical agent has antibacterial properties, silver ions have been reportedly found in patients who received sulfadiazine silver, and this is associated with negative outcomes after burns or with pressure ulcers.^[30]^

Despite the encouraging therapeutic effects of the combined group on pressure ulcers, our study still had several limitations. First, the number of patients that were included in this study is limited, leading to a lower statistical power. Moreover, the evolution of bacterial microbiota is needed to provide dynamic changes in the composition of the microbiota species in the wound. Besides, conventional management of pressure ulcers could be included as the control group to discuss the advantages and shortcomings of our treatment arms.

**Figure 1.**
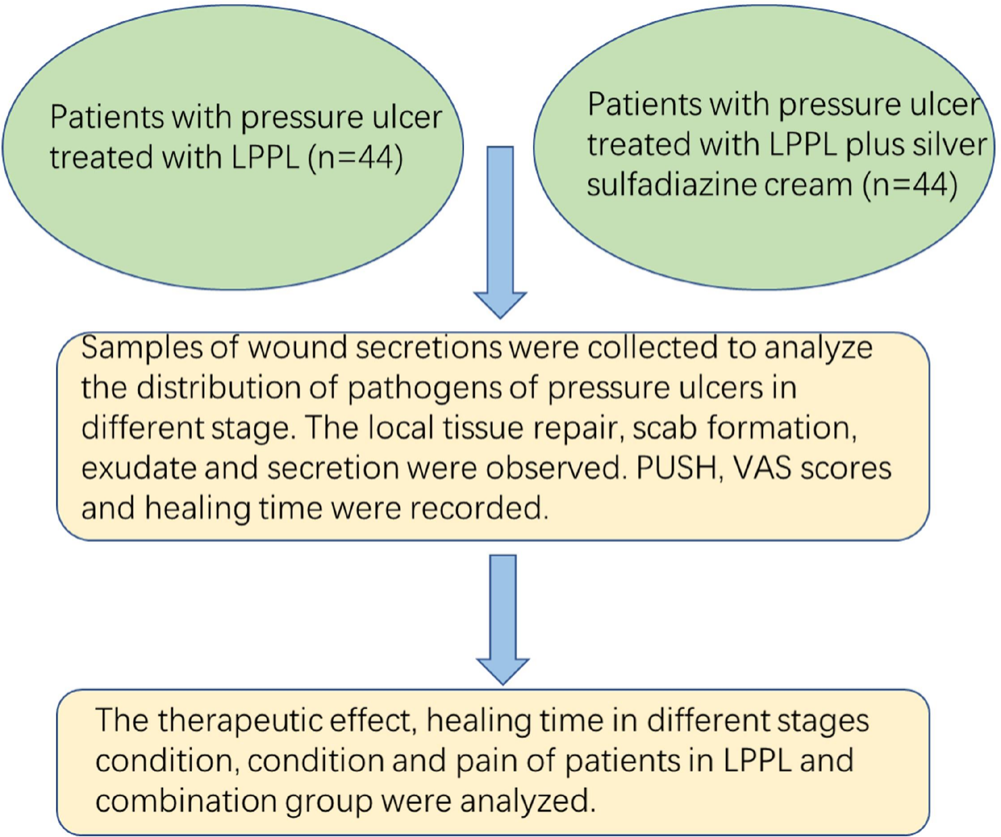
Clinical observation of LPPL combined with silver sulfadiazine cream in the treatment in different stages of pressure ulcers. LPPL = linear polarized polychromatic light.

To sum up, infections in pressure ulcer wounds are mainly caused by Gram-negative bacteria, based on routine nursing. LPPL combined with silver sulfadiazine cream has beneficial effects on the treatment of stage II to IV pressure ulcers, which can promote wound healing, thereby reducing patient’s suffering. Due to its simple and feasible application, this combination treatment is worth popularizing and its clinical use is a possible option in the future (Fig. 1).

## Author contributions

**Conceptualization:** Binxiong Chen.

**Data curation:** Yang Liu.

**Funding acquisition:** Yueming Liu, Shi Xu.

**Investigation:** Yueming Liu, Shi Xu.

**Methodology:** Yang Liu.

**Supervision:** Yueming Liu.

**Writing – original draft:** Binxiong Chen.

**Writing – review & editing:** Shi Xu.
